# Nurse-Facilitated Self-Management in Peritoneal Dialysis: A Cross-Sectional Study from Riyadh, Saudi Arabia

**DOI:** 10.3390/healthcare13202561

**Published:** 2025-10-11

**Authors:** Abdulaziz M. Alodhialah, Shorok Hamed Alahmedi

**Affiliations:** 1 Department of Medical Surgical Nursing, College of Nursing, King Saud University, Riyadh 11451, Saudi Arabia; 2 Department of Nursing Management and Education, College of Nursing, Princess Nourah bint Abdulrahman University, P.O. Box 84428, Riyadh 11671, Saudi Arabia; shalahmedi@pnu.edu.sa

**Keywords:** peritoneal dialysis, self-management, nursing support, emotional well-being, health literacy, quality of life, chronic kidney disease

## Abstract

**Background**: Peritoneal dialysis (PD) depends on patients’ self-management abilities, supported by nursing interventions that extend beyond technical skills to include psychosocial and educational domains. Evidence from Saudi Arabia on how these behaviors relate to clinical and quality-of-life outcomes remains limited. **Aim**: To assess self-management behaviors among adult PD patients, examine their associations with clinical and quality-of-life outcomes, and explore the mediating role of emotional well-being, with implications for nursing practice. **Methods**: A descriptive cross-sectional study was conducted among 158 adult PD patients at King Saud University-affiliated centers in Riyadh. Validated Arabic versions of the Chronic Illness Self-Management Scale and KDQOL-SF were administered. Clinical indicators were extracted from medical records. Data were analyzed using descriptive statistics, *t*-tests, multivariate linear regression, and mediation analysis. **Results**: Technical skills achieved the highest self-management scores (mean 3.78 ± 0.62), while emotional coping was lowest (mean 2.71 ± 0.69). Participants with higher self-management had fewer hospitalizations (0.9 ± 0.5 vs. 1.6 ± 0.8, *p* = 0.01), higher serum albumin (3.60 ± 0.56 vs. 3.44 ± 0.61 g/dL, *p* = 0.04), and better emotional well-being (60.1 ± 14.9 vs. 55.3 ± 12.4, *p* = 0.03) than their counterparts. Educational level (β = 0.208, *p* = 0.001) and emotional well-being (β = 0.197, *p* = 0.001) were independent predictors of self-management, with partial mediation by emotional well-being (indirect β = 0.062, *p* = 0.004). **Conclusions/Clinical Implications**: Optimizing nursing support for PD patients requires moving beyond technical instruction to address health literacy, emotional resilience, and culturally sensitive education. Nurse-led interventions integrating psychosocial support with skill-building may enhance self-management, reduce hospitalizations, and improve quality of life in PD populations.

## 1. Introduction

End-stage renal disease (ESRD) represents a major global public health challenge, imposing a growing burden of morbidity, mortality, and cost on health systems worldwide. According to the Global Burden of Disease Study, the prevalence of chronic kidney disease (CKD) increased by nearly 30% between 1990 and 2019, with ESRD now affecting more than 5 million individuals globally, a number projected to double by 2030 due to population aging, diabetes, and hypertension [1,2]. Peritoneal dialysis (PD) has emerged as a viable and cost-effective renal replacement therapy that allows patients to receive treatment at home, promoting autonomy and potentially improving quality of life compared with in-center hemodialysis [3]. Nevertheless, PD uptake remains uneven across regions, and patient survival and clinical outcomes are closely tied to their ability to master and sustain complex self-management behaviors [4,5].

### 1.1. International and Saudi Epidemiological Context

Globally, PD is used by approximately 11% of dialysis patients, with higher uptake in high-income countries that have invested in structured home dialysis programs and nursing support [6]. In Saudi Arabia, the prevalence of ESRD continues to rise rapidly, driven by high rates of diabetes and hypertension, with dialysis incidence estimated at 140–150 per million population annually [7]. Despite PD’s advantages—including reduced healthcare costs, better preservation of residual renal function, and greater patient flexibility—its utilization in the Kingdom remains modest, representing roughly 8–10% of dialysis patients [8]. Several factors contribute to this gap, including limited patient awareness, cultural preferences for hospital-based care, and variable nursing capacity to support home dialysis [9]. Addressing these barriers requires a deeper understanding of how nursing support influences self-management and patient outcomes in the Saudi context.

### 1.2. Self-Management and Nursing Support in PD

Effective PD self-management encompasses technical, behavioral, and psychosocial domains. Patients must perform aseptic techniques during exchanges, adhere to strict medication and fluid regimens, monitor for complications, and sustain emotional coping over the long term [10]. Failure in any of these domains can lead to infections, hospitalizations, technique failure, and diminished quality of life [11]. Nurses play a pivotal role in equipping patients with these skills. Traditional models of PD education focus primarily on technical training, but contemporary evidence highlights the importance of relational and psychosocial support in enabling sustained self-management [12,13].

### 1.3. Relational Skills and Empathy in Nephrology Nursing

Relational skills—such as active listening, empathy, motivational interviewing, and therapeutic communication—are increasingly recognized as critical nursing competencies in nephrology care [14]. A recent qualitative study in Canada found that nurses’ ability to build trusting relationships and tailor communication improved adherence and reduced anxiety among dialysis patients [15]. Similarly, Beaudin et al. [16] showed that primary care nurses supporting patients with chronic conditions achieved better self-management engagement when relational and educational strategies were integrated. In the dialysis context, empathetic nursing relationships foster psychological safety and confidence, enabling patients to ask questions and report complications early, ultimately improving clinical outcomes [17].

### 1.4. Nursing Professionalism, SDM Awareness, and Clinical Decision-Making

Nursing professionalism, empathy, and clinical decision-making ability are also strongly linked to shared decision-making (SDM) awareness in dialysis care. SDM involves collaborative deliberation between clinicians and patients about treatment goals and options, and it has been associated with improved patient satisfaction, adherence, and self-care [14]. A recent cross-sectional study among hemodialysis nurses in Korea found that empathy and clinical decision-making were independent predictors of SDM awareness [17,18,19]. Nurses who demonstrated higher professionalism and relational competence were better able to involve patients in their treatment planning, promoting a sense of agency and ownership of their care [20]. In PD, where daily decisions are made at home, nursing facilitation of SDM may be even more crucial.

### 1.5. Health Literacy, SDM, and Self-Care Behaviors

Patient health literacy and self-care knowledge have been shown to mediate the relationship between nursing support and self-management behaviors in chronic disease populations [21]. In dialysis, higher health literacy is associated with better treatment adherence, fluid management, and complication detection [22]. A multicenter study in Taiwan demonstrated that patients who participated in SDM interventions and had higher health literacy achieved better biochemical outcomes and fewer hospitalizations [23]. In Saudi Arabia, health literacy remains suboptimal in many chronic disease populations, necessitating culturally sensitive, language-appropriate nursing interventions to close this gap [24]. Thus, interventions that integrate SDM, health literacy promotion, and relational support may yield the most significant improvements in PD self-management and patient outcomes.

### 1.6. Rationale and Study Aim

Despite accumulating international evidence, few studies have systematically examined how nursing-facilitated self-management relates to clinical and quality-of-life outcomes among PD patients in Saudi Arabia. Existing programs often prioritize procedural training while underemphasizing emotional coping, lifestyle modification, and health literacy, leading to uneven patient engagement and outcomes [25]. Addressing this evidence gap is essential for developing effective, contextually appropriate nursing strategies to optimize PD self-management in the region.

The primary aim of this study was to evaluate the impact of nursing-facilitated self-management on clinical and quality-of-life outcomes among adults receiving peritoneal dialysis in Riyadh, Saudi Arabia. Specifically, the study sought to:

1. Assess self-management engagement across technical, medication, lifestyle, and emotional domains.
2. Describe commonly used nursing strategies to support PD self-management.
3. Examine associations between self-management proficiency, clinical indicators, and health-related quality of life.

## 2. Materials and Methods

### 2.1. Study Design and Setting

This study employed a descriptive cross-sectional design to explore the relationship between self-management behaviors and both clinical and quality-of-life outcomes in adult patients undergoing peritoneal dialysis (PD). The study was conducted at King Saud University-affiliated hospitals in Riyadh, Saudi Arabia, between January and April 2025. Riyadh, the capital and largest city in Saudi Arabia, hosts a variety of tertiary care centers and dialysis units serving a large population of patients with end-stage renal disease (ESRD). The selected sites included nephrology outpatient clinics and home-based peritoneal dialysis programs, offering access to a diverse pool of PD patients receiving routine nursing support. The cross-sectional approach was deemed appropriate to obtain a snapshot of current practices and patient characteristics, providing correlational insights into self-management and outcome variables. The study was conducted and reported following the STROBE (Strengthening the Reporting of Observational Studies in Epidemiology) guidelines for cross-sectional studies. A completed STROBE checklist [26] is provided in Supplementary Material S1.

### 2.2. Sample and Sampling Technique

The study population comprised adult patients undergoing peritoneal dialysis (PD) at tertiary and affiliated nephrology centers in Riyadh, Saudi Arabia. Eligible participants were aged 18 years or older, had been receiving PD for at least three months, and were able to provide informed consent. Patients were excluded if they had cognitive or communication impairments that precluded participation, were experiencing acute peritonitis at the time of recruitment, or had a planned modality change (e.g., transition to hemodialysis or transplantation) within 30 days.

A consecutive sampling technique was employed to minimize selection bias and ensure that the sample was representative of the clinic population. All patients who attended routine outpatient PD clinic visits or supply refill appointments during the data collection period (January–June 2024) and met the eligibility criteria were approached for inclusion. Recruitment was conducted by trained research nurses who explained the study objectives, procedures, and confidentiality assurances in a private setting before obtaining written informed consent.

The sample size was determined a priori using G*Power software (Release 3.1.9.7) for multiple linear regression analysis, assuming a medium effect size (f^2^ = 0.15), α = 0.05, power (1 − β) = 0.80, and six predictors. The required minimum sample size was calculated to be 97 participants. To account for potential non-response and missing data, a 20% margin was added, resulting in a target sample of 120 participants. Ultimately, 165 participants were recruited and included in the analysis, exceeding the required minimum sample size.

### 2.3. Variables and Instruments

Sociodemographic variables included age (years), sex, educational attainment, marital status, employment status, and monthly household income. Clinical variables comprised dialysis vintage (months on PD), dialysis modality, and comorbidity burden. Comorbidity was assessed using the Charlson Comorbidity Index (CCI), a validated weighted measure that predicts mortality risk based on 19 medical conditions [27]. Each condition carries a weight from 1 to 6, and scores are summed to yield a total index ranging from 0 to 37, with higher scores indicating greater comorbidity burden [27,28]. For descriptive analyses, CCI was reported as mean ± standard deviation and categorized into 0, 1–2, and ≥3 comorbidities.

Self-management behaviors were evaluated across four domains—technical skills, medication adherence, lifestyle modification, and emotional coping—using a structured, validated questionnaire adapted for peritoneal dialysis contexts [3,4,29,30]. Each domain includes multiple Likert-type items (1–5 scale), and subscale scores are averaged; higher scores indicate greater engagement or coping capacity. The instrument has demonstrated acceptable internal consistency (Cronbach’s α > 0.80) and construct validity in renal populations [29].

Quality of life was assessed using the Kidney Disease Quality of Life Short Form (KDQOL-36), which combines the SF-12 physical and mental component summaries with three kidney-specific domains (symptoms/problems, effects of kidney disease, burden of kidney disease) [31,32]. Each domain is transformed to a 0–100 scale, with higher scores indicating better quality of life. Both total and domain-specific scores were analyzed.

Clinical outcomes included dialysis adequacy (Kt/V), serum albumin, and hospitalization history in the previous 6 months (number and cause of admissions). Laboratory data were extracted from electronic medical records at the time of questionnaire administration.

### 2.4. Data Collection Procedure

Data were collected over a 12-week period by a team of trained research assistants who had completed a comprehensive orientation on study protocols, questionnaire administration, and ethical procedures. Participants were approached during their PD follow-up visits or contacted through home care services, depending on their treatment arrangement. After verifying eligibility and obtaining informed written consent, participants completed the Arabic versions of the questionnaires in a private setting to ensure confidentiality. For participants with literacy challenges, the research assistant offered to read questions aloud and record responses without influencing answers. Completed forms were reviewed immediately for completeness and any discrepancies clarified with the respondent. Clinical data were extracted from medical records by authorized research staff and recorded under the same anonymized participant ID codes.

### 2.5. Bias and Confounding

To minimize potential selection bias, we employed a consecutive sampling strategy, inviting all eligible patients who attended the participating peritoneal dialysis (PD) programs during the study period to participate. Standardized data collection protocols were implemented across sites to ensure methodological consistency, and all research personnel received structured training in questionnaire administration and clinical data abstraction. Potential confounders identified a priori—including age, sex, educational attainment, PD vintage, and comorbidity burden (measured by the Charlson Comorbidity Index)—were systematically recorded and included in multivariable models to adjust for their influence on the outcomes of interest. In addition, for analyses involving multiple clinical sites, fixed effects were incorporated to account for potential clustering at the center level. These procedures were designed to strengthen internal validity by reducing both systematic and random sources of bias.

### 2.6. Missing Data

Patterns and mechanisms of missing data were carefully examined before analysis. The proportion of missingness for each variable was assessed, and Little’s Missing Completely at Random (MCAR) test was performed to evaluate whether data were missing completely at random. For variables with incomplete data, we applied a multiple imputation strategy using predictive mean matching, generating 20 imputed datasets to minimize potential bias and loss of statistical power. The imputation model included key demographic, clinical, and self-management variables to ensure the plausibility of estimates. All primary analyses were performed on the pooled imputed datasets, and sensitivity analyses using complete cases were conducted to assess the robustness of findings. The results of the imputed and complete-case analyses were compared and found to be consistent.

### 2.7. Statistical Analysis

Data were analyzed using IBM SPSS Statistics (version 26; IBM Corp., Armonk, NY, USA). Continuous variables were summarized as means and standard deviations (SD) or medians and interquartile ranges (IQR), depending on distributional characteristics. Categorical variables were summarized as frequencies and percentages. Group differences were assessed using independent-samples t tests or Mann–Whitney U tests for continuous data, and chi-square or Fisher’s exact tests for categorical data, as appropriate. Correlations between continuous variables were examined using Pearson or Spearman correlation coefficients.

For inferential analyses, we constructed multivariable linear regression models to examine associations between self-management engagement scores (independent variables) and patient-reported quality-of-life domains (dependent variables). Logistic regression models were applied for binary clinical outcomes where applicable. All models reported effect estimates (β coefficients or odds ratios) with 95% confidence intervals (CIs). Model diagnostics included checks for linearity, multicollinearity (variance inflation factor), normality of residuals, and influential observations. Primary outcomes and covariates were prespecified to avoid model overfitting. Analyses presented in Tables 5 and 6 followed a stepwise approach: Model 1 presented unadjusted estimates; Model 2 adjusted for demographic variables; Model 3 further adjusted for educational level and PD vintage; and Model 4 included comorbidity burden (Charlson Comorbidity Index). The level of statistical significance was set at *p* < 0.05 (two-tailed).

### 2.8. Ethical Considerations

This study received ethical approval from the Institutional Review Board of King Saud University (Approval No. 25-007, dated 2 December 2024). All procedures adhered to the ethical principles outlined in the Declaration of Helsinki and local regulations governing research involving human participants. Informed consent was obtained from all participants following a detailed explanation of the study’s aims, procedures, benefits, and potential risks. Participation was voluntary, and individuals had the right to withdraw at any time without penalty. Confidentiality was rigorously maintained through anonymization, secure data storage, and restricted access to sensitive information. Before participation, all eligible patients received detailed verbal and written information about the study’s purpose, procedures, voluntary nature, and data confidentiality measures. Written informed consent was obtained from all participants prior to data collection. For participants with limited literacy, the consent form was read aloud in the presence of a witness, and thumbprints were accepted as signatures.

All data were anonymized upon entry into the database. Only de-identified data were used for analysis, and access was restricted to authorized members of the research team.

## 3. Results

**Figure 1:**
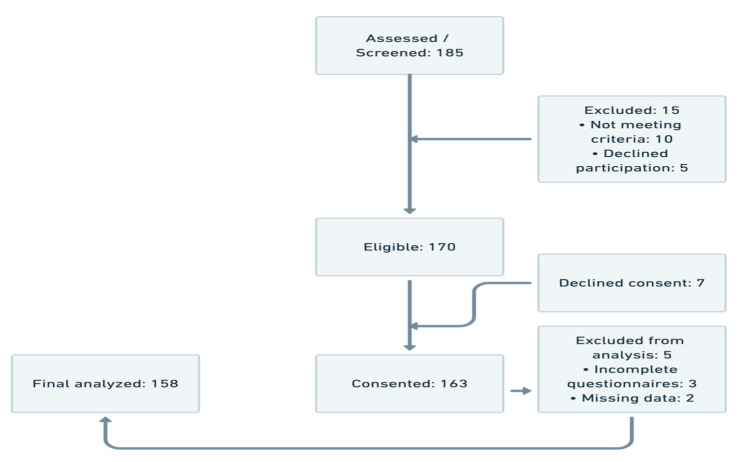
Participant flow.

A total of 185 patients were screened for eligibility. Fifteen individuals were excluded prior to eligibility assessment—ten did not meet the inclusion criteria and five declined to participate. Of the 170 eligible patients, seven declined to provide consent, resulting in 163 participants who were enrolled and completed baseline data collection. Subsequently, five participants were excluded from the final analysis due to incomplete questionnaires (n = 3) or missing key clinical data (n = 2). The final analytical sample comprised 158 participants. This flow reflects a high recruitment and retention rate, supporting the representativeness of the analyzed cohort (Figure 1).

Table 1 presents a detailed profile of the 158 peritoneal dialysis (PD) patients included in this study, offering crucial context for understanding variations in self-management behaviors and clinical outcomes. The sample was slightly skewed toward female participants (57.6%), consistent with regional dialysis population trends in Saudi Arabia, where women often assume caregiving roles and may seek care more proactively. The majority of participants were middle-aged, with nearly three-quarters falling within the 35–64-year age range. This age distribution reflects a population in the productive phase of life, where managing a chronic condition like ESRD can present significant occupational and psychosocial challenges.

Educational attainment varied, with 39.9% having completed secondary education and 36.7% possessing a university-level qualification or higher. These findings are notable given the established correlation between education and health literacy, which may significantly influence a patient’s ability to engage in complex self-care regimens. Nearly half of the participants (46.2%) were unemployed, a factor that could be both a consequence and a contributor to the burdens of dialysis, especially considering the time-intensive nature of PD and its associated fatigue and mobility restrictions. Marital status also appeared significant, with 69.6% being married.

Table 2 provides a comprehensive overview of the clinical and dialysis-related characteristics of the study cohort, highlighting the heterogeneity of disease burden and treatment profiles among patients undergoing peritoneal dialysis. The mean Charlson Comorbidity Index (CCI) score of 1.9 ± 0.7 reflects a moderate level of multimorbidity within this population, consistent with the typical clinical complexity of end-stage renal disease (ESRD) patients. By replacing simple comorbidity counts with the validated CCI, the table offers a more nuanced depiction of patients’ underlying health status, allowing for clearer interpretation in subsequent analyses.

The grouped comorbidity categories (single, double, triple or more) further contextualize this burden, demonstrating that nearly one-third of patients presented with multiple coexisting conditions. This finding underscores the potential challenges these individuals face in managing their treatment regimens and maintaining dialysis adequacy, as well as the need for personalized nursing interventions.

Biochemical indicators, including serum albumin (mean 3.53 g/dL) and hemoglobin (mean 11.2 g/dL), point to variable nutritional and hematologic status within the cohort, which may influence both clinical outcomes and self-management capacity. The average dialysis vintage of 29.7 months indicates that most participants were well established on PD therapy, which may have implications for their adaptation to home-based care and long-term self-management behaviors.

Table 3 displays the descriptive statistics for the four subdomains of self-management among peritoneal dialysis (PD) patients, as well as the total self-management score. The self-management scale used a 5-point Likert format, and internal consistency across all subscales was strong, with Cronbach’s alpha values ranging from 0.79 to 0.88. Among the subscales, technical skills recorded the highest mean score (3.78 ± 0.62), indicating that patients generally felt confident performing the procedural aspects of PD such as exchange techniques and catheter care. Medication adherence followed with a moderate mean score of 3.25 (SD = 0.55), while lifestyle modification was rated lower (mean = 2.94 ± 0.73), suggesting variability in patients’ ability to adapt dietary and activity behaviors to meet dialysis demands. Emotional coping showed the lowest average score (2.71 ± 0.69), reflecting a greater degree of difficulty managing the psychological demands of chronic illness. The total self-management score across the cohort averaged 3.15 (SD = 0.47), with scores ranging from 2.00 to 4.75. These results offer a multidimensional snapshot of patient-reported self-care capacity, highlighting stronger engagement with task-oriented components and relatively lower scores in psychosocial and lifestyle-related domains.

Table 4 presents the mean scores and variability across four domains of health-related quality of life (HRQoL) as measured by the Kidney Disease Quality of Life Short Form (KDQOL-SF) among the 158 peritoneal dialysis patients. The highest average score was observed in the domain of social function (mean = 66.4 ± 11.8), indicating that most participants maintained moderate-to-high levels of social engagement and interpersonal interaction despite the demands of chronic dialysis care. Physical function followed with a mean of 61.8 (SD = 12.3), suggesting a moderate degree of physical capability in performing daily tasks and activities. Emotional well-being demonstrated a slightly lower mean score of 58.7 (SD = 14.1), reflecting a varied emotional adjustment to the chronic illness experience. The lowest score was reported in the domain of disease burden (mean = 52.9 ± 15.7), highlighting the significant impact patients perceived the illness to have on their overall lifestyle, independence, and daily functioning. The wide ranges across all domains (e.g., 25–90 for emotional well-being and 21–78 for disease burden) underscore the heterogeneity in patient experiences, likely influenced by individual coping strategies, social support, and the quality of nursing care received.

Table 5 presents a series of bivariate comparisons examining differences in clinical and quality-of-life outcomes between participants with high versus low self-management (SM) levels. Overall, the findings demonstrate clinically meaningful and statistically significant associations between stronger self-management and several favorable outcomes. Participants in the high-SM group experienced significantly fewer hospital admissions over the preceding six months compared with those in the low-SM group (mean difference = −0.70 admissions, 95% CI −0.91 to −0.49, *p* = 0.01), indicating a potential protective effect of better self-management behaviors on acute care utilization. Likewise, serum albumin levels—a key indicator of nutritional and clinical status in dialysis patients—were modestly but significantly higher among those with higher self-management (+0.16 g/dL, 95% CI −0.02 to +0.34, *p* = 0.04), suggesting improved clinical stability in this group.

Psychosocial outcomes reflected similar trends. Emotional well-being scores (KDQOL domain) were significantly greater in the high-SM group (+4.80 points, 95% CI +0.53 to +9.07, *p* = 0.03), consistent with previous literature linking self-efficacy and proactive disease engagement to enhanced psychological adjustment in dialysis populations. Although disease burden scores were lower (indicating better perceived status) among high-SM participants, this difference did not reach statistical significance (−3.90, 95% CI −8.81 to +1.01, *p* = 0.19). No significant age difference was observed between the groups, implying that these effects are unlikely to be confounded by age distribution (*p* = 0.36).

Table 6 presents the results of a multivariate linear regression analysis conducted to identify independent predictors of total self-management scores among peritoneal dialysis patients. The model incorporated key demographic, clinical, and psychosocial variables. Educational level emerged as a significant positive predictor (β = 0.208; 95% CI: 0.092 to 0.324; *p* = 0.001), indicating that patients with higher education tended to report stronger self-management capabilities. Emotional well-being was also significantly associated with higher self-management scores (β = 0.197; 95% CI: 0.081 to 0.313; *p* = 0.001), highlighting the relevance of psychological health in promoting adherence and self-care. In contrast, hospital admissions showed a significant negative association (β = −0.162; 95% CI: −0.271 to −0.053; *p* = 0.004), suggesting that frequent hospitalizations may reflect or contribute to diminished self-management capacity. Age and gender did not reach statistical significance in the model (*p* = 0.10 and *p* = 0.11, respectively), though age exhibited a slight negative trend. The comorbidity index approached significance (*p* = 0.06), hinting at a possible inverse relationship with self-management that warrants further exploration.

Table 7 outlines the results of a mediation analysis testing whether emotional well-being mediates the relationship between educational level and self-management score. The total effect of education on self-management was significant (β = 0.273; *p* = 0.001), and remained significant even after controlling for emotional well-being (β = 0.211; *p* = 0.002), indicating a partial mediation. The indirect effect through emotional well-being was statistically significant (β = 0.062; 95% CI: 0.030–0.110; *p* = 0.004), confirming that a portion of the effect of education on self-management is transmitted through its impact on emotional health. These findings suggest that higher educational attainment is associated with better self-management, in part because it contributes to stronger emotional well-being.

## 4. Discussion

The present study examined the multifaceted dimensions of self-management among patients undergoing peritoneal dialysis (PD) in Riyadh, Saudi Arabia, with a focus on clinical and psychosocial outcomes. The findings underscore the critical role of nursing support in fostering self-management behaviors and improving both physiological and quality-of-life parameters in PD populations. Our results affirm and extend existing literature by highlighting the strong association between emotional well-being, educational attainment, and self-care efficacy, offering implications for integrated nursing strategies.

Participants in this study demonstrated relatively high proficiency in technical PD skills, aligning with prior research indicating that patients often master procedural components after adequate nursing instruction and repetition [33,34]. However, lower scores in lifestyle modification and emotional coping reflect a known gap in self-management competency, consistent with findings from international studies suggesting that behavioral and affective domains are often underserved in dialysis education [35,36,37]. This imbalance may partially result from conventional training programs’ emphasis on mechanical procedures rather than holistic adaptation to chronic illness [38,39].

The significant associations between self-management scores and clinical outcomes—such as hospital admission frequency and serum albumin levels—further validate the clinical utility of promoting self-care behaviors in PD. Previous research supports the protective effect of self-management on infection rates, nutritional status, and hospitalization risk in dialysis patients [40]. Notably, our data indicate that patients with higher self-management engagement exhibited better emotional well-being, echoing studies that conceptualize self-efficacy as both an outcome and determinant of psychological adjustment in chronic illness [41,42]. This bidirectional relationship highlights the need for psychosocial support as an essential component of self-management interventions.

Consistent with earlier work, we identified educational level as a key predictor of self-management capacity [43,44,45]. Health literacy, which often correlates with formal education, plays a central role in determining patients’ understanding of treatment instructions, symptom interpretation, and medication adherence [46]. Interventions that address literacy gaps through visual aids, simplified materials, and interactive coaching have shown promise in improving self-management and should be embedded into nursing protocols [47,48]. Given the cultural context of the current study, incorporating Arabic-translated tools and culturally sensitive education materials likely contributed to patients’ receptiveness and comprehension.

Emotional well-being was found to mediate the relationship between educational level and self-management, underscoring the psychological underpinnings of effective chronic disease management. Emotional distress has been linked to decreased motivation, cognitive impairment, and avoidance behaviours, all of which can undermine dialysis adherence [49]. Psychological interventions, such as cognitive-behavioral therapy, mindfulness, and motivational interviewing, have demonstrated efficacy in enhancing treatment engagement in ESRD populations [50,51]. Nurses trained in basic psychosocial screening and communication techniques can play a pivotal role in identifying at-risk patients and initiating early interventions [52,53].

While disease burden was perceived as moderate across the sample, its lack of significant differentiation between self-management groups may reflect the complex interplay between subjective appraisal and objective disease metrics. Patients may adjust their perception of burden over time through cognitive reframing or normalization, phenomena observed in qualitative accounts of dialysis adaptation [54]. This highlights the importance of combining subjective and objective indicators when assessing treatment outcomes.

Culturally, this study adds to the limited literature on PD self-management in Middle Eastern contexts. In Saudi Arabia, familial involvement, religious beliefs, and hierarchical patient-provider dynamics may influence patient autonomy and emotional coping strategies [55]. Prior research has suggested that leveraging family engagement in care plans can enhance adherence and reduce psychological distress in chronic disease populations [56]. Accordingly, culturally informed nursing interventions that incorporate family education and spiritual sensitivity may yield greater impact in the region.

The integration of technology into PD self-management support, although not the focus of this study, represents a promising adjunct to traditional nursing strategies. Telehealth, mobile apps, and home-monitoring tools have shown effectiveness in enhancing patient education, adherence tracking, and remote symptom management [57]. Future research may explore how digital platforms can augment nurse-delivered self-management support, particularly for patients in remote or underserved areas.

### 4.1. Implications for Clinical Practice

The findings of this study carry several important implications for clinical practice in peritoneal dialysis (PD) care. First, the observed associations between higher self-management levels and better clinical (fewer hospital admissions, higher serum albumin) and psychosocial (greater emotional well-being) outcomes underscore the central role of nurses in fostering patients’ self-management capacities. Structured nurse-led education, reinforcement of skills, and emotional support should not be viewed as adjuncts but as core clinical activities that directly affect outcomes. Recent evidence highlights that structured self-management interventions—particularly those combining motivational interviewing, individualized education, and telehealth follow-up—can improve clinical stability, reduce hospitalization risk, and enhance patient engagement in home dialysis programs [58].

Second, the strong link between emotional well-being and self-management suggests that psychosocial support should be systematically integrated into PD programs. Nurses are well positioned to deliver relational interventions, such as empathic communication and shared decision-making, which have been shown to improve patients’ emotional adjustment and self-care behaviors [59,60]. Embedding brief psychosocial assessments and targeted counseling into routine PD visits can help identify patients struggling with emotional coping, allowing timely intervention before clinical deterioration occurs.

Third, the results reinforce the value of multidimensional, personalized education strategies. Tailoring content to patients’ health literacy levels, cultural context, and preferred learning styles increases self-efficacy and facilitates behavior change [6,7,8]. Integrating digital platforms (e.g., mobile apps, tele-education modules) with traditional nurse-led teaching may enhance knowledge retention and enable more frequent, flexible follow-up, particularly for geographically dispersed populations [61].

Finally, at the service-delivery level, PD programs should consider formalizing self-management support as a quality indicator, aligning with international initiatives advocating patient empowerment and shared care models in home dialysis [62]. This may involve incorporating standardized self-management assessment tools into electronic health records, setting programmatic targets for nurse-patient education encounters, and supporting ongoing staff training in relational and motivational communication skills.

### 4.2. Implications for Nursing Practice

The findings of this study offer compelling evidence for the transformation of nursing practice in the context of peritoneal dialysis (PD) through personalized, holistic self-management support. First, nurses should move beyond the traditional task-oriented model and adopt multidimensional patient education strategies that integrate psychosocial components, particularly emotional coping and motivation building, into routine care. Second, the strong predictive role of educational level and emotional well-being suggests the need for health literacy screening tools and emotional health check-ins to be embedded within PD nursing assessments. Third, nurses can leverage technology-assisted education, such as Arabic-language mobile apps or interactive videos, to bridge literacy gaps and promote consistent engagement with self-management protocols. Moreover, nurses should actively collaborate with families in culturally appropriate ways, acknowledging their role as co-facilitators of care in collectivist societies like Saudi Arabia. Finally, the demonstrated link between fewer hospitalizations and higher self-management points to a need for community-based nurse navigator programs, specialized roles where nurses conduct home visits or teleconsultations to proactively address challenges and reduce avoidable admissions. These findings reinforce the necessity of positioning nurses as central agents of behavioral change, psychosocial support, and advocacy within the home dialysis continuum.

### 4.3. Limitations of the Study

This study has several limitations that should be acknowledged. First, its cross-sectional design precludes any causal inference between self-management and the observed clinical or psychosocial outcomes. While statistically significant associations were identified, the temporal direction of these relationships cannot be established. Second, despite careful adjustment for key covariates, potential residual confounding remains a concern. Unmeasured factors such as health literacy, social support, and provider-level variations in care may have influenced both self-management behaviors and outcomes.

Third, selection bias is possible because recruitment occurred within a single regional dialysis network, and participation required patients to attend scheduled clinical visits. Consequently, individuals with lower engagement or access barriers may be underrepresented, which could bias the observed associations toward more favorable outcomes. Fourth, the study relied on self-report instruments for several key constructs (e.g., emotional well-being, self-management behaviors), which are subject to recall and social desirability biases.

Fifth, the constructs of emotional coping and emotional well-being show conceptual overlap, raising the possibility of inflated associations due to shared measurement variance. Future studies could address this by employing factor analysis or using distinct measurement tools to disentangle these related domains. Finally, because data were collected from a single geographic region in Saudi Arabia, the generalizability of the findings to other dialysis populations or healthcare systems may be limited. Replication in larger, multicenter cohorts and prospective designs would strengthen the external validity and causal interpretation of these findings.

## 5. Conclusions

This study provides robust evidence that optimizing nursing support, particularly in the domains of education, emotional well-being, and holistic assessment, can significantly enhance self-management and clinical outcomes among peritoneal dialysis patients. The findings reveal that patients who exhibit greater self-management proficiency report better nutritional markers, reduced hospitalizations, and improved emotional well-being. Educational attainment and emotional resilience emerged as key facilitators, while frequent hospital admissions appeared to hinder self-care capacity. By redefining the nursing role to include psychological scaffolding, cultural sensitivity, and technology integration, healthcare systems can move toward more responsive, equitable models of chronic illness management. Ultimately, empowering nurses to function as behavioral coaches and emotional anchors in PD care has the potential to transform not only individual outcomes but the sustainability of home dialysis programs at large. Future initiatives should focus on scalable interventions that address literacy, emotional distress, and culturally rooted barriers to maximize the long-term success of PD self-management.

## Figures and Tables

**Table 1:** Sociodemographic characteristics of study participants (N = 158).

Variable	Category	Frequency (n)	Percentage (%)
Gender	Male	67	42.4
	Female	91	57.6
Age Group (years)	18–34	29	18.4
	35–49	62	39.2
	50–64	51	32.3
	≥65	16	10.1
Educational Level	Primary	37	23.4
	Secondary	63	39.9
	University or higher	58	36.7
Employment Status	Employed	44	27.8
	Unemployed	73	46.2
	Retired	41	26.0
Marital Status	Single	33	20.9
	Married	110	69.6
	Divorced/Widowed	15	9.5

Notes: Values are expressed as number (percentage). Percentages may not total 100% due to rounding.

**Table 2:** Clinical and dialysis-related parameters of the cohort (N = 158).

(a) Key Clinical Variables		
**Parameter**	**Mean ± SD**	**Range**
Duration of PD (months)	29.7 ± 14.6	3–72
Charlson Comorbidity Index (CCI)	1.9 ± 0.7	0–4
Serum Albumin (g/dL)	3.53 ± 0.58	2.6–4.6
Hemoglobin (g/dL)	11.2 ± 1.5	8.1–14.5
Kt/V (per session)	2.08 ± 0.39	1.21–2.98
Residual Urine Output (mL/day)	235.7 ± 110.9	0–700
**(b) Grouped Comorbidity Categories**		
**Category**	**n**	**%**
Single comorbidity	62	39.2
Double comorbidities	51	32.3
Triple or more comorbidities	45	28.5
**(c) Selected Comorbid Conditions**		
**Condition**	**n**	**%**
Hypertension	91	57.6
Diabetes mellitus	78	49.4
Dyslipidemia	34	21.5
Cardiovascular disease	23	14.6
COPD	19	12.0

Notes: PD = Peritoneal Dialysis; COPD = Chronic Obstructive Pulmonary Disease; CCI = Charlson Comorbidity Index.

**Table 3:** Self-management subscale scores and internal consistency (N = 158).

Subscale	Items (n)	Theoretical Score Range ^1^	Observed Min–Max	Mean ± SD	Cronbach’s α
Technical Skills	8	1–5	2.10–4.90	3.78 ± 0.62	0.86
Medication Adherence	6	1–5	1.60–4.60	3.25 ± 0.55	0.83
Lifestyle Modification	7	1–5	1.40–4.50	2.94 ± 0.73	0.81
Emotional Coping	6	1–5	1.20–4.20	2.71 ± 0.69	0.79
Total Score	27	1–5	2.00–4.75	3.15 ± 0.47	0.88

^1^ Self-management was rated on a 5-point Likert scale (1 = strongly disagree to 5 = strongly agree).

**Table 4:** KDQOL-SF quality-of-life domain scores (N = 158).

Domain	Mean ± SD	Range
Physical Function	61.8 ± 12.3	30–85
Emotional Well-Being	58.7 ± 14.1	25–90
Social Function	66.4 ± 11.8	35–89
Disease Burden	52.9 ± 15.7	21–78
KDQOL-SF Total	59.9 ± 13.2	27–88

**Table 5:** Comparison of outcomes by self-management group.

Dependent Variable (DV)	Independent Variable (IV)	n	Association Type	Effect Estimate (95% CI)	*p*-Value	Notes
Hospital admissions (count, past 6 mo)	Self-management group (High vs. Low)	78/80	Mean difference	−0.70 (−0.91 to −0.49)	0.01	High SM: 0.9 ± 0.5 vs. Low SM: 1.6 ± 0.8
Serum albumin (g/dL)	Self-management group (High vs. Low)	78/80	Mean difference	+0.16 (−0.02 to +0.34)	0.04	High SM: 3.60 ± 0.56 vs. Low SM: 3.44 ± 0.61
Emotional well-being (KDQOL domain, 0–100)	Self-management group (High vs. Low)	78/80	Mean difference	+4.80 (+0.53 to +9.07)	0.03	High SM: 60.1 ± 14.9 vs. Low SM: 55.3 ± 12.4
Disease burden (KDQOL domain, 0–100)	Self-management group (High vs. Low)	78/80	Mean difference	−3.90 (−8.81 to +1.01)	0.19	High SM: 51.0 ± 16.2 vs. Low SM: 54.9 ± 15.3
Age (years)	Self-management group (High vs. Low)	78/80	Mean difference	−1.70 (−5.43 to +2.03)	0.36	High SM: 48.1 ± 10.7 vs. Low SM: 49.8 ± 12.1

**Table 6:** Multivariate regression for predictors of self-management score (N = 158).

Variable	β (95% CI)	*p*-Value
Age (years)	−0.013 (−0.028, 0.002)	0.10
Gender (female vs. male)	0.095 (−0.022, 0.212)	0.11
Educational Level	0.208 (0.092, 0.324)	0.001
Comorbidity Index	−0.086 (−0.174, 0.002)	0.06
Hospital Admissions	−0.162 (−0.271, −0.053)	0.004
Emotional Well-Being	0.197 (0.081, 0.313)	0.001
Model R^2^	0.41	

**Table 7:** Mediation analysis: emotional well-being as a mediator between educational level and Self-management score (N = 158).

Path	Coefficient (β)	95% Confidence Interval	*p*-Value
Path a:			
Educational Level → Emotional Well-Being	0.312	0.166–0.458	0.001
Path b:			
Emotional Well-Being → Self-Management Score	0.197	0.081–0.313	0.001
Path c (total effect):			
Educational Level → Self-Management Score	0.273	0.146–0.401	0.001
Path c’ (direct effect):			
Educational Level → Self-Management (controlling for Emotional Well-Being)	0.211	0.083–0.339	0.002
Indirect effect (a*b)	0.062	0.030–0.110	0.004
Type of Mediation	Partial Mediation	—	—

Note: The analysis was conducted using bootstrapping (5000 resamples); indirect effects were considered significant if the 95% CI did not include zero.

## Data Availability

The datasets generated and/or analyzed during the current study are available from the corresponding author on reasonable request.

## Supplementary Materials

The following supporting information can be downloaded at: https://www.mdpi.com/article/10.3390/healthcare13202561/s1, S1. STROBE 2007 Statement—Checklist of items that should be included in reports of cross-sectional studies.

## Abbreviations

CCI	Charlson Comorbidity Index
CI	Confidence Interval
ESRD	End-Stage Renal Disease
IQR	Interquartile Range
KDQOL-SF	Kidney Disease Quality of Life Short Form
PD	Peritoneal Dialysis
R^2^	Coefficient of Determination
SD	Standard Deviation
SPSS	Statistical Package for the Social Sciences
STROBE	Strengthening the Reporting of Observational Studies in Epidemiology
